# Genetic studies on the APOA1-C3-A5 gene cluster in Asian Indians with premature coronary artery disease

**DOI:** 10.1186/1476-511X-7-33

**Published:** 2008-09-19

**Authors:** Jayashree Shanker, Ganapathy Perumal, Veena S Rao, Natesha B Khadrinarasimhiah, Shibu John, Sridhara Hebbagodi, Manjari Mukherjee, Vijay V Kakkar

**Affiliations:** 1 Mary and Garry Weston Functional Genomics Unit, Thrombosis Research Institute, Bangalore, India; 2 Tata Proteomics & Coagulation Unit, Thrombosis Research Institute, Bangalore, India; 3 Elizabeth & Emmanuel Kaye Bioinformatics and Statistics Unit, Thrombosis Research Institute, Bangalore, India; 4 Thrombosis Research Institute, London, UK

## Abstract

**Background:**

The APOA1-C3-A5 gene cluster plays an important role in the regulation of lipids. Asian Indians have an increased tendency for abnormal lipid levels and high risk of Coronary Artery Disease (CAD). Therefore, the present study aimed to elucidate the relationship of four single nucleotide polymorphisms (SNPs) in the Apo11q cluster, namely the -75G>A, +83C>T SNPs in the APOA1 gene, the Sac1 SNP in the APOC3 gene and the S19W variant in the APOA5 gene to plasma lipids and CAD in 190 affected sibling pairs (ASPs) belonging to Asian Indian families with a strong CAD history.

**Methods & results:**

Genotyping and lipid assays were carried out using standard protocols. Plasma lipids showed a strong heritability (h^2 ^48% – 70%; *P *< 0.0001). A subset of 77 ASPs with positive sign of Logarithm of Odds (LOD) score showed significant linkage to CAD trait by multi-point analysis (LOD score 7.42, *P *< 0.001) and to Sac1 (LOD score 4.49) and -75G>A (LOD score 2.77) SNPs by single-point analysis (*P *< 0.001). There was significant proportion of mean allele sharing (pi) for the Sac1 (pi 0.59), -75G>A (pi 0.56) and +83C>T (pi 0.52) (*P *< 0.001) SNPs, respectively. QTL analysis showed suggestive evidence of linkage of the Sac1 SNP to Total Cholesterol (TC), High Density Lipoprotein-cholesterol (HDL-C) and Apolipoprotein B (ApoB) with LOD scores of 1.42, 1.72 and 1.19, respectively (*P *< 0.01). The Sac1 and -75G>A SNPs along with hypertension showed maximized correlations with TC, TG and Apo B by association analysis.

**Conclusion:**

The APOC3-Sac1 SNP is an important genetic variant that is associated with CAD through its interaction with plasma lipids and other standard risk factors among Asian Indians.

## Background

Lipids and lipoproteins have been traditionally associated with high risk of incident coronary artery disease (CAD). The Apolipoprotein A1-C3-A4-A5 gene cluster on chromosome 11q23 (Apo11q) is among the most well characterized regions of the human genome with reference to their dynamic association with plasma lipids and lipoproteins [1]. The associated haplotypes constitute a highly informative genetic marker [2]. Extensive interactions both within and between the genetic variants within this cluster contribute to the quantitative variation in the blood lipid phenotypes [3]. The NCBI db SNP Build 116 has established over 182 single nucleotide polymorphisms (SNPs) and 4 ins/del variants in this genetically rich region [4]. A complex pattern of gene expression has been demonstrated through in vivo studies, wherein the Apo CIII enhancer acts as a common regulatory element for the APOA1-C3-A4 genes but not for the APOA5 gene [5]. Consistent association has been reported from population-based studies [6,7] as well as in families with familial combined hyperlipidemia (FCHL) [8,9] and in individuals with hypertriglyceridemia [10] across populations from Americas [9,11], Europe [12,13] and Asia [14,15]. Both linkage and association analysis on FCHL families have shown that genetic variation at the Apo11q gene cluster contribute to the transmission of FCHL phenotype across generations in these families [9]. Although many studies have shown a strong association between the genetic variants in the Apo11q region with plasma lipid phenotypes, there have been a few reports to the contrary [16,17], implying genetic heterogeneity.

The SNPs in the Apo11q region, particularly the 3238C>G, Sac1 SNP in the 3' untranslated region (UTR) of the APOC3 gene [18], as well as -1131 C>T promoter SNP and S19W SNP in exon 2 of the APOA5 gene [10,19] have been shown to independently and in tandem, regulate plasma TG levels [13]. The two promoter SNPs, -75G>A and +83C>T in the APOA1 gene, have shown strong association with HDL-C and ApoA1 levels [20]. Although individual genetic variants within the Apo11q gene cluster have been independently associated with CAD in some studies [21,22], a majority of them have not been able to establish such a direct association [23-26].

Association studies have been previously reported on the Asian Indian population, mostly on healthy adult volunteers, to elucidate the role of specific genetic variants in the APOA1 gene [27], APOC3 gene [28] or the APOA5 gene [29] in regulating plasma lipid levels. Given that this population has an inherent tendency for dyslipidemia and associated high risk of CAD [30], the objective of the present study was to estimate linkage and association between the 4 genetic variants, namely APOA1, -75G>A and +83C>T SNPs, the APOC3, Sac1 SNP and the APOA5, S19W SNP in the Apo11q gene cluster with premature CAD as well as with lipids and lipoproteins in a cohort of affected siblings belonging to Asian Indian families with strong history of CAD. Additionally, we have attempted to elucidate the relationship between circulating lipids and conventional coronary risk factors in our cohort.

## Methods

### Study participants

A total of 523 families comprising 2318 individuals were enrolled in Phase1 of the Indian Atherosclerosis Research Study [IARS], an ongoing genetic epidemiological study that aims to identify specific genes and regulatory pathways associated with CAD in the Asian Indian population. Families were identified through a proband with a strong family history of CAD and/or stroke, from select hospitals/clinics in Bangalore and Mumbai. Probands were defined as patients with angiographically proven ischemic heart disease with age at onset ≤ 60 years for men and ≤ 65 years for women. Other affected/unaffected family members (parents, siblings, spouse and offspring over 18 years of age) were recruited when available and willing. None of the participants had a history of past major illness or concomitant infection. The institutional ethics committee approved the IARS, which was conceived according to the guidelines defined by the Indian Council of Medical Research for research conducted on human subjects. A signed informed consent was obtained from all participants in the study.

### Questionnaire

A detailed case record form pertaining to information on demographics, anthropometry, medical history and coronary risk factors such as presence of diabetes, hypertension, smoking, lifestyle and current medication was completed for each participant through personal interviews and through perusal of their medical records.

### Blood sampling

Venous blood was collected in evacuated tubes after an overnight fast of 12 to 14 hours (Vacuette^®^, Greiner Bio-One GmbH, Vienna, Austria). Serum, EDTA and citrate plasma samples were separated by centrifugation and aliquots were preserved at -80°C until analysis. Cell pellets were stored at -20°C and used for extraction of genomic DNA at a later date.

### Laboratory investigations

Serum Total cholesterol (TC), Triglycerides (TG), Lipoprotein (a) (Randox Laboratories Ltd, UK), High Density Lipoprotein-cholesterol (HDL-cholesterol) (Bayer Diagnostics, Randox Laboratories, Dade-Behring Ltd, UK), Apolipoprotein A1 (Apo A1) and Apolipoprotein B100 (Apo B) (Orion Diagnostics, Finland) were estimated on the Cobas Fara II Clinical Chemistry Auto analyzer (F. Hoffman La Roche Ltd., Switzerland), following the manufacturer instructions. Serum Low Density Lipoprotein-cholesterol (LDL-cholesterol) was calculated using the Friedwald's formula. Three commercial controls purchased from Randox Laboratories, one from Orion Diagnostica for apolipoproteins and a normal in-house serum pool were run with every batch of assay. The inter-assay coefficients of variation (CV) for the commercial controls and normal serum pool ranged from 4.9% to 7.0% for TC, 6.1% to 7.7% for TG, 7.1% to 12.2% for HDL-cholesterol, 9.9% to 14.2% for ApoA1 and 10.7% to 13.9% for ApoB levels.

### Heritability of lipid traits

Heritability refers to the proportion of phenotypic variance attributable to genetic variance. Heritability estimates were performed for the various atherothrombotic phenotypes in 508 IARS families consisting of 2305 individuals. Circulating levels of lipid markers were measured in family units comprising proband, spouse and offspring. Age- and sex-adjusted heritability was estimated by variance component analysis using the sequential oligogenic analysis routine (SOLAR) (SOLAR v 1.4.1) program. Spouse pair correlations were determined to account for the household effects.

### Genetic studies on the APOA1-C3-A5 gene cluster

**Figure 1:**
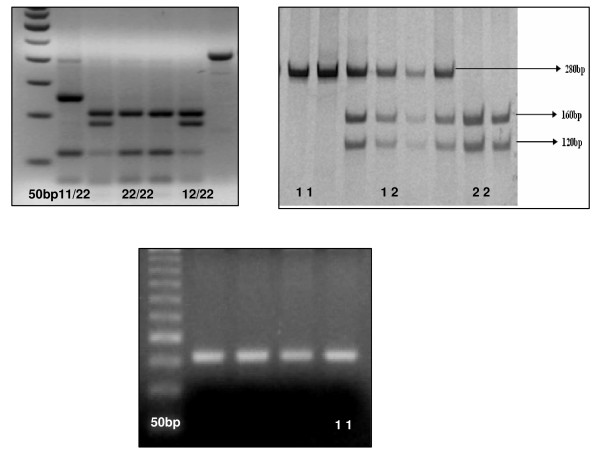
Representative gels of the 4 SNPs in the APOA1-C3-A5 gene cluster.

Genomic DNA was extracted by the salting-out procedure [31]. Based on the published reports of a strong association between the 3238 C>G Sac I SNP (rs5128) in the 3'UTR of the APOC3 gene and plasma TG levels, a preliminary linkage study was conducted on 190ASPs. Subsequently, two promoter SNPs, -75G>A (rs1799837) and +83C>T (rs5069) in the APOA1 gene and the S19W SNP (rs3135506) in exon 2 of the APOA5 gene were also selected for genotyping in this study. Details of PCR primers, annealing conditions and restriction enzyme digestion have been described elsewhere [10,32,33]. In short, the genomic regions encompassing the polymorphic sites were PCR amplified using site-specific primers, restriction digested with appropriate restriction enzymes at 37°C overnight and the digests were resolved on 2% agarose gel for the APOA1 (Figure. 1A) and the APOA5 SNPs (Figure. 1C) while the APOC3 genotypes were resolved by 6% polyacrylamide gel electrophoresis (PAGE) (Figure. 1B). The Gene amp PCR System 2700 (Applied Biosystems, CA, USA) was used for PCR amplification and gels were stained with ethidium bromide and visualized by ultraviolet trans-illuminator. Images capture and analysis was performed on DOC-IT and Lab works-4 software (UVP Ltd. Cambridge, UK), respectively. All genotypes were checked three times independently using appropriate heterozygotes as positive controls.

### Affected sibling pairs [ASPs]

The present study consisted of 194 ASPs selected from 131 IARS families. Of these, there were 114 families containing 1 ASP, 14 with 3 ASPs and two families with 6 ASPs, respectively, while one family consisted of 15 ASPs. Taking into account the genotyping failures in some cases, only data on 190 ASPs for whom genotyping was performed for all the four SNPs, was used in the final analysis.

### Linkage analysis

Non-parametric linkage analysis was performed by the ASP analysis method [34] to test for linkage between CAD and the four SNPs across the Apo11q gene cluster.

For the preliminary study, linkage between the Sac1 SNP and plasma TG levels was tested using the deviation in the proportions of 0-, 1- or 2- alleles shared, identity by state (IBS) between the 190 ASPs, against an expected distribution of 25%, 50% and 25%, respectively. Association of the Sac1 genotypes with plasma TG levels was tested by analysis of variance (data unpublished).

Three additional SNPs (ApoA1 -75G>A, +83C>T and the APOA5, S19W) were used for the subsequent linkage study. Appropriate input files for linkage analysis were created with the MEGA2 program [35]. Multipoint and single point linkage analysis was performed using MERLIN_all _and MERLIN_pairs _options under MERLIN [36] as well as the LODPAL option in S.A.G.E v. 5.3.1 package. Significant linkage was assigned based on the criteria of Kruglyak et al [37]: 0 = no linkage; 1 = suggestive linkage; 2 = significant linkage; 3 = highly significant linkage; and 4 = confirmed linkage. Linkage and mean proportion of alleles shared (pi), identity by descent (IBD), was tested for each of the markers across the sib pairs. A pi that was greater than 0.50 was considered as significant. The GENIBD program in SAGE was used to run IBD analysis.

### Subset analysis

On the basis of the individual sign of LOD score obtained from linkage results on the entire dataset (N = 287), families where all ASPs showed a positive LOD score (LOD score ≥ 0) (N = 104) were selected for further analysis [38]. Additionally, families were classified based on the following characteristics i.e. families having at least one affected sibling in an ASP with age of onset less than 45 years (N = 108) and 'hyper TG families' where one (N = 162) or both sibling pairs (N = 46) had TG values ≥160 mg/dL. Linkage and IBD allele sharing was individually calculated for the whole dataset and the subset groups.

### Quantitative trait loci [QTL] analysis

Analysis of QTL was performed on families with positive sign of LOD score and 'hyper TG families' using the 'QTL' option in MERLIN program. All lipid variables (TC, TG, HDL-cholesterol, LDL-cholesterol, ApoA1 and ApoB) were independently tested against the four markers for evidence of linkage. Age, gender, smoking, diabetes, hypertension and statin use were used as covariates.

### Association studies- statistical methods

Routine statistical analysis was performed using the SPSS v.15 (Statistical program for social sciences) package. Mean and standard error of mean (SEM) were calculated for the quantitative variables. Genotype frequencies were determined by direct counting. Conformity to Hardy-Weinberg equilibrium was determined by χ^2 ^test (*P *> 0.05). The major allele was referred to as 1 and the minor allele as 2; the corresponding genotypes were assigned as 1 1, 1 2 or 2 2 for the homozygous normal, heterozygotes and homozygous variants, respectively. Genetic variants with the 2 2 genotypes, having a frequency of less than 10 (Apo A1 +83C>T and Apo AV S19W) were grouped with the heterozygotes for analysis purpose. Haplotype frequency was estimated using the DECIPHER program of SAGE.

Correlations among the plasma lipids and lipoproteins were tested using the Pearson's correlation coefficients estimate. Student t-test or ANOVA and multivariate analyses were used to test for association between the Apo11q SNPs and plasma levels of lipids and lipoproteins before and after adjusting for confounding factors such as age, gender, BMI, smoking, presence of diabetes or hypertension and statin use. Step-wise linear regression analysis was employed to understand the extent of contribution of individual SNPs to the variation in plasma lipid and lipoprotein levels. The non-parametric Kolmogorov-Smirnov test was used as test of normality for the quantitative variables and the values were subsequently log-transformed to normalize distribution. CAD severity was defined on the basis of age at CAD onset, the number of diseased vessels and the event score (1 = ischemic heart disease with chest pain, 2 = stable angina, 3 = unstable angina/MI) classified according to the clinician's diagnosis. The Chi-square test was used to test for association between genotypes and CAD severity. Other discrete variables such as presence/absence of diabetes, hypertension and smoking were also tested against the 4 Apo11q SNPs. Presence of diabetes (Type 2 DM) and hypertension (HTN) was ascertained based on self-report of physician's diagnosis and/or use of prescription medications along with proof obtained from their medical records. A nominal *P *value of 0.05 or less was considered as statistically significant.

### Canonical correlation statistics

In order to determine the contribution of SNPs to the variation in lipid markers in a cohort of subjects with premature CAD, a two-stage procedure was adopted comprising of correspondence analysis in the first stage followed by canonical correlations [39]. The 'R' statistical package was used for this analysis. Correspondence analysis is an exploratory, multi-dimensional scaling technique that is applied to discrete variables, wherein the row and column weightages are obtained for maximum discrimination in the form of a contingency table of categorical data. Here, the linear functions of the row effects would best differentiate the columns and vice versa.

The set of discrete variables consisted of the four SNPs in the Apo11q cluster, hypertension and diabetes. In the first stage, these categorical data were subjected to correspondence analysis in order to obtain the scores for each row category represented by the individual subjects in the CAD group that were derived as linear functions of the column profiles characterized by the respective genotypes for the 4 SNPs, hypertension and diabetes. These provide the least square estimates obtained as a linear function, which act as weightages of each individual.

In the second stage, Canonical correlation analysis was performed between the quantitative variables namely the lipid markers, body mass index (BMI) and waist hip ratio (WHR) (Set 1 variables) and the coordinates derived by correspondence analysis carried out in the 1^st ^stage (Set 2 variables). Canonical correlation analysis is a multivariate data analysis technique, which maximizes the correlations between the linear functions of two sets of variables. Since canonical correlations are best defined when both the sets are continuous in nature, we adopted this two-stage procedure.

## Results

A total of 531 families were recruited in the Phase 1 of the IARS, of which 23 incomplete families were excluded from analysis. Of these, 131 families having 190 ASPs were selected for the present study. Gender-wise distribution of lipid profile, CAD severity and coronary risk factors among the affected siblings is shown in Table 1. There were more males (84.7%) than female (15.3%). Although the mean levels of lipid markers were higher among the females than males, significant differences were observed only for TG (*P *= 0.046) and ApoA1 (*P *= 0.013). Diabetes (*P *= 0.006) and hypertension (*P *= 0.004) were significantly higher among females (67.4%, 72.1%) when compared to the corresponding proportions in males (44.9%, 48.2%). While 47.3% of males were smokers, there were no female smokers in our cohort. Mean age of CAD onset was 50.3 ± 8.4 years for males and 53 ± 8.8 years for females. The number of diseased vessels and event score showed similar distribution in both sexes. More than 93% of females were postmenopausal but none of them were receiving hormone replacement therapy. Over 62.6% of males and 78.3% of females were on the lipid lowering drug, statin, respectively.

**Table 1:** Distribution of the various phenotypic variables in males and females.

**DESCRIPTIVES**	**MALES (N = 243)**		**FEMALES (N = 44)**	
	
	**(%)**	**Mean ± SD**	**(%)**	**Mean ± SD**
Age at recruitment		57.2 ± 8.7		57.82 ± 7.59
Body Mass Index (kg/m^2^)		25.63 ± 3.8		28.38 ± 5.4
Waist-Hip-Ratio		0.96 ± 0.05		0.87 ± 0.09
Diabetes	44.9		67.4	
Hypertension	48.2		72.1	
Smoking	47.3		-	
Total cholesterol		156.73 ± 39.3		166.23 ± 36.72
Triglycerides		149.39 ± 75.3		150.59 ± 52.02
High Density lipoprotein		37.86 ± 9.49		39.91 ± 8.75
Low Density Lipoprotein		88.88 ± 33.29		96.17 ± 35.43
Apolipoprotein A1		1.12 ± 0.24		1.18 ± 0.18
Apolipoprotein B100		0.92 ± 0.26		0.93 ± 0.20
Age at onset of CAD		51.34 ± 8.6		53.64 ± 7.8
Event score				
1 (Mild)	3.9		4.8	
2 (Moderate)	27.6		28.6	
3 (Severe)	68.4		66.7	
No. of disease vessels				
1	16.9		23.3	
2	21.9		30.0	
≥ 3	61.2		46.4	
Statin therapy	62.6		78.3	
Beta blockers	55.7		69.6	
ACE inhibitors	32.8		41.3	
Calcium Channel blockers	37.3		23.9	
Hypoglycemic agents	32.8		36.9	

CAD, coronary artery disease.

### Heritability of lipid traits

Significant heritability (*P *< 0.0001) was observed for TC (h^2 ^= 0.65 ± 0.06), TG (h^2 ^= 0.53 ± 0.06), HDL-cholesterol (h^2 ^= 0.48 ± 0.06), LDL-cholesterol (h^2 ^= 0.55 ± 0.07), Apo A1 (h^2 ^= 0.71 ± 0.04) and Apo B (h^2 ^= 0.70 ± 0.05). There was minimal spouse-pair correlation for most lipid traits (r = 0.02 – 0.09; *P *> 0.05) except HDL-cholesterol, which showed a higher correlation coefficient value (r = 0.26; *P *< 0.001).

### Linkage analysis of the Apo11q markers and CAD

**Figure 2:**
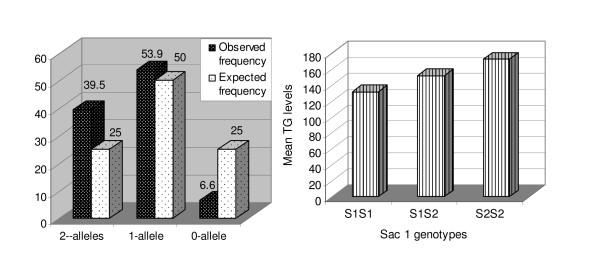
**A. Mean Plasma TG Levels (mg/dl) across the APOC3 Sac1 genotypes. B.** Proportion of mean alleles shared across the APOC3-Sac 1 genotypes.

Preliminary studies on the APOC3-Sac1 polymorphism and plasma TG levels on 190 ASPs showed that the proportion of 0-, 1- and 2-alleles shared IBS, between the ASPs was 6.3%, 38.9% and 54.8%, respectively, which deviated significantly from the expected proportion of 25%, 50% and 25% allele sharing (*P *< 0.0001), suggesting tentative linkage of the Sac-1 locus with CAD (Figure 2A). Significant difference was observed in the mean TG levels (137.61 ± 4.35 mg/dl, 151.77 ± 6.95 mg/dl and 172.33 ± 11.72 mg/dl) (*P *< 0.0001) across the APOC3-Sac1 genotypes, namely S1S1, S1S2, and S2S2, respectively (Figure 2B).

Based on the suggestive linkage of the APOC3-Sac1 locus to CAD and the association of minor allele with plasma TG levels, this study was subsequently extended to include three additional SNPs in the Apo11q gene cluster. No evidence of linkage was observed by both multipoint and single point linkage analysis as well as by IBD analysis for any of the 4 SNPs in the whole data set. However, linkage analysis on a subset of 47 families with 77 ASPs showing a positive LOD score sign (LOD ≥ 0), gave highly significant LOD score of 7.42 (*P *< 0.001) by multipoint analysis for the CAD trait, which was confirmed using the LODPAL program of SAGE. Single point analysis showed strong evidence of linkage for the Sac 1 SNP (LOD score 4.49; *P *< 0.0001) and a suggestive evidence of linkage for the -75G>A SNP (LOD score 2.77; *P *= 0.0002). The mean proportion of alleles shared IBD was significant for the Sac-1 (pi 0.59; *P *< 0.0001), -75G>A (pi 0.56; *P *< 0.0001) and +85C>T (pi 0.52; *P *= 0.0012) SNPs using the GENIBD program of SAGE. (Table 2)

**Table 2:** Findings of linkage analysis.

**Analysis Method**	**Whole data** **(ASPs = 190)**	**Subset Data (Positive LOD score families) (ASPs = 77)**		
		
		**Sac1 LOD score** **(*P *value)**	**-75G>A**	**+83C>T**
Multipoint Linkage analysis	NS	7.42 (<0.001)		
Single Point Linkage	NS	4.49 (<0.0001)	2.77 (0.0002)	NS
IBD Pi (*P *value)	NS	Pi 0.59 (<0.0001)	Pi 0.56 (<0.0001)	Pi 0.52 (0.0012)
QTL	NS	TC 1.42 (0.005) HDL-c 1.72 (0.002) ApoB 1.19 (0.010)	NS	NS

ASPs, Affected sib pairs; IBD, identity by descent; Pi, mean proportion of allele sharing; NS, Not significant, QTL, Quantitative trait loci.

Ordered subset analysis performed on ASPs, who were selected based on age at onset < 45 years and plasma TG levels, did not show any evidence of linkage.

### Quantitative trait loci analysis

The APOC3-Sac 1 SNP showed suggestive evidence of linkage to TC (LOD score 1.42; *P *= 0.005), HDL-cholesterol (LOD score 1.72; *P *= 0.002) and Apo B (LOD score 1.19; *P *= 0.010) only in families with positive LOD score (Table 2).

When the various coronary risk factors as well as CAD severity were compared between the group with positive sign of LOD score (N = 104) and negative sign of LOD score (N = 181), the mean levels of the various lipid markers were marginally higher among the former group than the latter group. The difference was statistically significant only for plasma ApoA1 (*P *= 0.024) levels. Other features such as CAD severity, BMI, WHR, and frequency of Type 2 DM and HTN showed similar distribution across the two groups (Data not shown).

### SNPs in the Apo11q gene cluster

All the four SNPs in the Apo11q cluster were in Hardy Weinberg equilibrium (*P *> 0.05). The distribution of minor allele frequencies (MAF) and genotypes across the four SNPs is given in Table 3. While the Sac-1 (0.32), the -75G>A (0.19) and the 85C>T (0.15) SNPs were highly polymorphic, the S19W SNP exhibited a low MAF (0.03). Haplotype analysis using the PHASE program in SAGE showed that the haplotypes with a frequency greater than 0.10 for the four SNPs, -75G>A, +83 C>T, Sac-1 and S19W were 1-1-1-1 (0.40), 1-1-2-1(0.32) and 2-1-1-1 (0.15), respectively.

**Table 3:** Minor allele frequency and genotype distribution across the four Apo11q SNPs.

**SNP**	**MAF**	**GENOTYPE**		
		
		**1 1** **n (%)**	**1 2** **n (%)**	**2 2** **n (%)**
**-75G>A (rs1799837)**	0.19	182 (65)	79 (31)	13 (4)
**+83C>T (rs5069)**	0.15	249 (90)	27 (10)	
**Sac1 (rs5128)**	0.32	132 (46)	111 (44)	32 (10)
**S19W (rs3135506)**	0.03	270 (94)	17 (6)	

MAF, minor allele frequency; SNP, single nucleotide polymorphism.

### Genotype-phenotype analysis

Association between the Apo11q genotypes and quantitative phenotypes were performed on the whole dataset of 190 ASPs comprising of 287 CAD subjects. Significant correlation was observed among the lipid and lipoprotein biomarkers using the Pearson's correlation coefficient estimates (Table 4). While correlations between TC, TG, LDL-cholesterol, ApoA1 and ApoB (r = 0.16 – 0.92; *P *< 0.001) were positive, there was significant negative correlation between the HDL-cholesterol and TG levels (r = -0.22; *P *< 0.001). This trend was retained following covariate adjustment for age, gender and BMI.

**Table 4:** Correlation coefficients between the lipid variables

	**TC**	**TG**	**HDL-c**	**LDL-c**	**ApoA**	**ApoB**
	**r (*P *value)**	**r (*P *value)**	**r (*P *value)**	**r (*P *value)**	**r (*P *value)**	**r (*P *value)**
**TC**	-	0.44 (<0.001)	0.21 (<0.001)	0.92 (<0.001)	0.40 (<0.001)	0.71 (<0.001)
**TG**	0.44 <0.001)	-	-0.22 (<0.001)	0.16 (0.007)	0.21 (<0.001)	0.53 (<0.001)
**HDL-c**	0.21 (<0.001)	-0.22 (<0.001)	-	0.05 (0.38)	0.39 (<0.001)	-.020 (.76)
**LDL-c**	0.92 (<0.001)	0.16 (0.007)	0.05 (0.38)	-	0.25 (<0.001)	0.61 (<0.001)
**ApoA1**	0.40 (<0.001)	0.21 (<0.001)	0.39 (<0.001)	0.25 (<0.001)	-	0.55 (<0.001)
**ApoB**	0.71 (<0.001)	0.53 (<0.001)	-0.02 (0.76)	0.61 (<0.001)	0.55 (<0.001)	-

ApoA1, apolipoprotein A1; ApoB, apolipoprotein B; BMI, Body mass index; HDL-c, high density- cholesterol; LDL-c, low density lipoprotein-cholesterol; TC, total cholesterol; TG, triglycerides

Multivariate analysis showed significant differences in the mean TG levels across the Sac-1 genotype (*P *= 0.007), with mean LDL-cholesterol levels across the -75G>A genotypes (*P *= 0.043) and with ApoA1 levels across the S19W genotypes (*P *= 0.026). Age was a common confounding factor that showed significant interaction with the lipids while gender and BMI did not make any significant impact in this dataset.

Results from linear regression analysis showed that APOC3-Sac1 SNP was the only significant contributory genetic marker among the 4 SNPs and was able to account for about 2.8% (*P *= 0.005) of the variation in plasma TG levels. With regard to the effect of conventional risk factors on lipids, HTN contributed to the variation in TC, TG, LDL-cholesterol, ApoA1 and ApoB levels (1.8% – 4.6%; *P *< 0.05), while waist hip ratio (WHR) contributed to over 1.5% (*P *= 0.037) of variation in HDL-cholesterol levels. Age was the other significant contributory factor to the variation among the plasma lipids (1.5% – 5.1%; *P *< 0.05).

### Estimation of maximized correlations

The results of correspondence analysis performed on the discrete variables namely the four SNPs, HTN and Type 2 DM indicated that the first three components accounted for nearly 79% of the total variability (Table 5). Based on standardized weightages, it was shown that the first component was characterized by the Sac1 (0.346) and -75G>A (0.308) SNPs, while the same two SNPs along with HTN (0.217) characterized the second component. Thus, these three traits were able to show maximum contribution to the variability in this CAD affected cohort. Structured correlation between these variables and their components indicated that the Sac1 SNP (0.844), the -75G>A SNP (0.639) and HTN (0.559) showed maximum contribution towards the first two components. Diabetes trait (0.915) was a significant contributor (p = 0.03) to the third component, which was able to extract around 18% of the total variability in this data set (Table 5).

**Table 5:** Correspondence analysis for discrete variables.

	**Component1**	**Component 2**	**Component 3**	**Component 4**	**Component 5**
Inertia	0.0407	0.0223	0.0185	0.0153	0.0069
**Cumulative Proportion**	**0.3924**	**0.6074**	**0.7862**	**0.9336**	**1**

***Coordinates***					

Sac1	**0.346**	**-0.142**	-0.031	-0.027	0.016
-75G>A	**-0.308**	**-0.216**	-0.03	-0.073	0.028
+ 83C>T	-0.054	0.057	-0.077	**0.274**	0.082
S19W	-0.039	0.017	-0.031	0.07	**-0.208**
HTN	-0.016	**0.217**	-0.133	**-0.136**	0.026
Type 2 DM	-0.011	0.081	**0.278**	-0.016	0.018

***Structured correlation***					

Sac1	**0.844**	**0.142**	0.007	0.005	0.002
-75G>A	**0.639**	**0.313**	0.006	0.036	0.005
+ 83C>T	0.031	0.035	0.063	**0.799**	0.072
S19W	0.03	0.006	0.019	0.096	**0.849**
HTN	0.003	**0.559**	0.21	**0.22**	0.008
Type 2 DM	0.002	0.077	**0.915**	0.003	0.004

HTN – hypertension; DM – Type 2 Diabetes mellitus.

The output of the canonical correlation analysis carried out between the Set1 variables comprising of the quantitative lipid traits and the Set 2 variables represented by the components of the Apo11q genotypes, HTN and Type 2 DM obtained by correspondence analysis are presented in Table 6, 7 and Table 7. Based on the R-square values, TG (0.081, p < 0.000), Apo B (0.079, p < 0.000) and TC (0.054, p < 0.025) were able to significantly contribute up to 21% towards the CAD trait. (Table 6). The standardized regression co-efficient data indicated that component 1 and component 2, characterized by the Sac-1 SNP, the -75 G>A SNP and HTN, were significantly associated with TG (0.154, p < 0.008) for the first component, TG (B = -0.147, p < 0.011) and ApoB (B = – 0.194, p < 0.001) for the second component while the component 3, characterized by DM, was associated with the Waist Hip Ratio (B = 0.117, p = 0.049) (Table 7). The regression coefficients between TG and ApoB with component 2 were observed to be negative. This is because, while calculating the least square estimates of weights of individual subjects by correspondence analysis, the components usually carry a +/- sign. The negative sign obtained while calculating the least square estimate values for the regression coefficients may be neglected, as they do not carry a physical implication. The output of the canonical correlation analyzed between the Set 1 variables and Set 2 variables are presented in Table 8. Here, the first two components accounted for up to 77% of the total variability (Table 8) and hence only these results are presented. The structured correlation between the lipid biomarkers and their components indicated that TG (r = 0.787), Apo B (r = 0.714) and TC (r = 0.469) contributed towards the first component, which accounted for over 52% of the maximized correlation, while HDL-cholesterol (R = 0.525) for the second component accounted for over 25% of the maximized correlations (Table 8). Like-wise, the structured correlation between the lipid biomarkers and the linear component of the second set of variables also indicated that the contributions of TG (R = 0.279), Apo B (R = 0.253) and TC (R = 0.166) were the three lipid biomarkers which were directly influenced by Sac 1 SNP, the -75 G>A SNP and hypertension in this cohort (Table 8).

**Table 6:** Squared multiple correlations

**Variables**	**R-Square**	**p value**
BMI (kg/m^2^)	0.023	0.266
WHR	0.021	0.321
TC (mg/dl)	**0.045**	**0.025**
TG (mg/dl)	**0.081**	**0.000**
HDL-c (mg/dl)	0.027	0.172
LDL-c (mg/dl)	0.037	0.065
Apo A1 (g/l)	0.031	0.114
ApoB (g/l)	**0.079**	**0.000**

ApoA1, apolipoprotein A1; ApoB, apolipoprotein B; BMI, Body mass index; HDL-c, high density- cholesterol; LDL-c, low density lipoprotein-cholesterol; TC, total cholesterol; TG, triglycerides

**Table 7:** Standardized regression coefficients and their probability values.

	**BMI** **r (p value)**	**WHR** **r (p value)**	**TC** **r (p value)**	**TG** **r (p value)**	**HDL-c** **r (p value)**	**LDL-c** **r (p value)**	**Apo A1** **r (p value)**	**ApoB** **r (p value)**
**Component 1**	-0.01 (0.097)	-0.003 (0.96)	0.071 (0.231)	**0.154 (0.008)**	-0.102 (0.085)	0.049 (0.403)	-0.016 (0.784)	0.071 (0.224)
**Component 2**	0.059 (0.32)	-0.048 (0.42)	-0.099 (0.091)	**-0.147 (0.011)**	0.022 (0.715)	-0.063 (0.284)	-0.106 (0.074)	**-0.194 (0.001)**
**Component 3**	0.051 (0.40)	**0.117 (0.05)**	-0.011 (0.848)	0.102 (0.077)	-0.079 (0.184)	-0.031 (0.603)	0.057 (0.335)	-0.011 (0.848)
**Component 4**	-0.037 (0.54)	0.066 (0.27)	**0.149 (0.011)**	**0.155 (0.008)**	-0.026 (0.661)	0.119 (0.045)	0.115 (0.052)	**0.188 (0.001)**
**Component 5**	-0.076 (0.20)	0.012 (0.837)	-0.083 (0.158)	-0.0003 (0.996)	0.097 (0.104)	**-0.122 (0.041)**	0.055 (0.353)	-0.003 (0.961)

ApoA1, apolipoprotein A1; ApoB, apolipoprotein B; BMI, Body mass index; HDL-c, high density- cholesterol; LDL-c, low density lipoprotein-cholesterol; TC, total cholesterol; TG, triglycerides.

**Table 8:** Findings of canonical correlation analysis.

	**Component 1**	**Component 2**
Canonical Correlation*	**0.355 (0.0001)**	0.253 (0.164)
Cumulative Proportion	**0.524**	**0.773**

**Table 7a. Standardized canonical coefficients for Set 1 Variables**		

BMI (kg/m^2^)	-0.4370	-0.2783
WHR	0.3720	-0.0457
TC (mg/dl)	-28.2070	-160.216
TG (mg/dl)	10.1196	53.7325
HDL-c (mg/dl)	6.6342	39.3671
LDL-c (mg/dl)	24.6045	139.0919
Apo A1 (g/l)	0.2284	-0.4109
Apo B (g/l)	0.2654	1.2400

**Table 7b. Structured correlations between Set 1 variables and their canonical variables**		

BMI (kg/m^2^)	-0.2682	-0.3134
WHR	0.2892	-0.1466
TC (mg/dl)	**0.4695**	-0.1716
TG (mg/dl)	**0.7874**	-0.1806
HDL-c (mg/dl	-0.2487	**0.5254**
LDL-c (mg/dl)	0.3015	-0.2712
Apo A1 (g/l)	0.4125	0.1872
Apo B (g/l)	**0.7138**	0.1761

**Table 7c. Structured correlations between Set 1 variables and the canonical variables of Set 2 variables**		

BMI (kg/m^2^)	-0.0952	-0.0794
WHR	0.1027	-0.0371
TC (mg/dl)	**0.1667**	-0.0435
TG (mg/dl)	**0.2795**	-0.0457
HDL-c (mg/dl)	-0.0883	0.1331
LDL-c (mg/dl)	0.1070	-0.0687
Apo A1 (g/l)	0.1464	0.0474
Apo B (g/l)	**0.2534**	0.0446

Set 1 Variables – BMI, WHR, TC, TG, HDL-c, LDL, ApoA1, ApoB; Set 2 variables – Components 1–5 obtained from correspondence Analysis; * p value for significance of canonical correlation coefficient provided in parenthesis

## Discussion

In the present study consisting of a cohort of affected sibling pairs selected from Asian Indian families with a strong history of premature CAD, the APOC3, Sac-1 and the ApoA1, -75G>A SNPs along with hypertension were the significant contributory factors to the CAD trait, probably mediated through their association with lipid and lipoprotein traits, particularly TC, TG and Apo B.

Additional evidence was obtained by linkage analysis in a subset of 77 ASPs who were selected based on positive sign of LOD score. Here, the Sac1 and the -75G>A SNPs showed significantly high LOD scores, implying linkage of these two loci to CAD as well as tentative linkage of the Sac1 SNP to TC, HDL-cholesterol and ApoB by QTL analysis. ASPs have been effectively used in linkage study, in families with cervical carcinoma, to identify several susceptible regions as well as a potential candidate gene underlying one of the loci on chromosome 9q32 region [40]. Despite a relatively low sample size (No. of ASPs = 278), the strength of their study was in the dense panel of over 500 microsatellite markers undertaken for their investigation.

In the present investigation, while tests of association have shown significant link between some of the Apo11q SNPs and lipid phenotypes in CAD siblings, the output from linkage analysis should be treated with some reservations, given the absence of linkage evidence in the whole dataset comprising of 190 ASPs. Although subset linkage analysis based on particular phenotypic traits such as lowered age of CAD onset or high TG levels within families has been generally popular in linkage studies [41], subset analysis based on positive LOD scores has been previously tested in schizophrenic patients [38] wherein they had shown that patients in the subset group exhibited a relatively severe clinical phenotype, which partly justified their selection criteria. In our study, however, no significant differences either pertaining to CAD severity or biomarker levels could be associated with the families with positive LOD score. Also, no evidence of linkage was observed in the other subsets analyzed such as age at onset less than 45 years or high TG levels. This may be attributed to the small size of ASPs and low number of SNPs genotyped in this study, which might have considerably reduced the power to detect significant linkage. Hence, validation on a larger number of sibling pairs is imperative before drawing definitive conclusions from these preliminary findings.

CAD is a complex, multifactor disease with strong interaction between the genes and environment [42]. In order to estimate the small contributions from individual genetic variants such as the SNPs or phenotypic traits such as the lipids towards the manifestation of a complex disorder like CAD calls for the application of rigorous statistical techniques. To this effect, we used two methods, namely correspondence analysis and canonical correlations, to analyze our data. While correspondence analysis groups the discrete variables into components based on their individuals weightage contributions and estimates the significant factors that contribute to the variability in the dataset, canonical correlations help to maximize the correlations between the linear functions of 2 sets of variables [39]. By correspondence analysis, we were able to show that the Sac-1 and -75G>A SNPs along with HTN belonging to the first two components were able to explain up to 62% of the variability among the 287 affected family members. On the other hand, these SNPs also showed significant association with TC, TG and ApoB traits. This indicates that the Apo11q variants might play a role in impacting CAD risk through their regulation of specific lipid biomarkers. A similar viewpoint has been reported by other investigators [43,44]. At this point it would be worthwhile to recall that the same two genetic variants and two of the lipid traits, TC and Apo B, showed significant linkage in subset analysis in this study.

Elevated plasma levels of TG and dense LDL-cholesterol particles along with low levels of HDL-cholesterol constitute an atherogenic lipoprotein phenotype [45]. That the genes in the Apo11q gene cluster is associated with lipid and lipoprotein phenotypes is well documented through family-based linkage studies [46] as well as genome-wide association studies [47], although the APOA1-C3-A4 region was able to explain less than 1% of the variation in the trait [47]. In the present study, of the 4 genetic markers, the APOC3-Sac1 SNP was the only variant that significantly contributed to about 2.8% of variation in plasma TG levels. The other SNPs, namely the -75G>A and the +83C>T SNPs, were significantly associated with plasma LDL-cholesterol and ApoA1 levels by multivariate analysis in our study. It has been previously shown that contributions from haplotypes rather than effects from single polymorphic variant within the cluster make a larger impact on the variation in circulating lipid levels [12].

The APOC3 Sac-1 polymorphism has been associated with CAD [4,21] and the Apo A1 -75G>A and the +83C>T SNPs have been reported to be a risk marker for CAD and MI among the Indian [27] and the Japanese subjects [48], respectively. Both the ApoA1 promoter SNPs are reported to be in strong linkage disequilibrium [22]. In the present study, both the Sac1 SNP and -75G>A SNP, the minor alleles in particular, showed significant association with CAD by correspondence analysis; however the +83C>T SNP with a very low MAF (0.03) did not add any significant value to our data analysis and findings. A similar low frequency of this allele has been previously reported among Asian Indians [29].

Environmental factors contribute significantly to the variation in plasma lipids [14]. While the IARS heritability data provided evidence for the strong influence of genetic factors for all the lipid traits, presence of small yet significant spouse-pair correlations reflected the effect of shared environmental risk factors in the IARS cohort. This was further confirmed through multivariate analysis, wherein, hypertension was an important factor that contributed between 1.8%–4.6%, of the variation in lipid levels. Further, hypertension was also a prominent feature of component 2 by correspondence analysis, contributing to over 22% of the variability in our dataset. In this regard, the report of an association between TG and the Apo11q gene cluster among hypertensive subjects from the BRIGHT study using genome wide approach is noteworthy [49]. Over 18% of the variability was attributed to Component 3, which was characterized by diabetes. Both hypertension and diabetes have been traditionally considered as important risk factors for CAD, especially among the Asian Indians [30]. Interestingly, diabetes also showed significant association with WHR based on standardized regression coefficients statistics. While there is a strong link between obesity and diabetes, the fact that WHR is a better marker of obesity than BMI has been reported previously by us [50] as well as others [51], particularly with reference to Asian Indians.

Over 49 SNPs have been determined in the 150 kb region spanning this gene cluster [52] and the number of variants has considerably increased following the addition of the APOA5 gene, 27 bp downstream to the Apo11q cluster [1]. The associated haplotypes within this genetically rich region constitute a highly informative genetic marker for the lipid trait [4]. Given this information, our study supports the notion that, the plasma lipids and lipoproteins, regulated partly by the genetic markers and their haplotypes within the Apo11q gene cluster, could serve as intermediary risk phenotypes that contribute to the pathophysiology of coronary artery disease.

While there is sufficient evidence for the association of genes in the Apo11q cluster with regard to circulating TG level, a recent report of a novel TG locus in the 1p31-32 region in Caucasian families with premature CAD/MI [53] strengthens the observation that triglyceride is an important risk marker for CAD, whose regulation may be under the genetic control of several chromosomal loci distributed across the genome. A large multiethnic study identified a variant, rs35136575 in another lipoprotein regulating loci, the apolipoprotein (APO) E/C1/C4/C2 cluster on chromosome 19, that accounted for 1% of variation in the LDL-cholesterol levels and exerted a pleiotropic, context-dependent effect on plasma lipoproteins [54]. The above findings point towards the complex processes that govern the expression of the genes regulating plasma lipid levels that may be under the joint influence of both genetic and environmental factors.

The present study has some shortcomings. Firstly, the size of our study population is small, which might not have had the power to detect evidence of linkage in the entire cohort of 190 ASPs. Secondly, only 4 markers were evaluated in a region spanning a large genomic distance of around 150 cM and is characterized by a dense array of SNPs, thereby reducing the probability of detecting the causal variant/s. Thirdly, association with other SNPs namely the ApoA1, +83C>T or APOA5, S19W was not observed probably due to a low MAF. Fourth, as previously mentioned, although the evidence from linkage analysis is encouraging, conducting subset analysis based only on siblings with positive sign of LOD score could lend bias to the findings. Finally, we are dealing with lipid traits whose levels are affected by statins. Over 65% of the subjects were on statin medication in this study. Although we have taken care to adjust for statin use in our analysis, we do not have information on the baseline lipid levels of these subjects to understand the proportion of cholesterol lowering effect that statins have on the circulating lipid levels.

On the other hand, there are several positive aspects to this study. The present cohort was enrolled from well-characterized families with a strong history of premature MI, which provide better opportunities to identify underlying association between the genetic markers and phenotypic traits in such a predisposed group. The IARS has shown strong heritability for plasma phenotypes, particularly the triglycerides, which prompted us to look into the Apo11q region. Our exploratory studies had initially shown strong association of the Sac-1 SNP to TG and tentative linkage to CAD by IBS analysis (data unpublished), which prompted us to expand by including additional markers in this region. Our hypothesis was strengthened by the findings of linkage analysis, albeit in a subset of the cohort. Application of sophisticated statistical analysis tool such as canonical correlations assisted in identifying and segregating the specific variables, namely the Sac1 SNP, -75G>A SNP and hypertension, that showed maximum contribution to the variability in our dataset of affected sibling pairs. The present study is to our knowledge, the first report to simultaneously look into the linkage and association of at least 4 markers in the APOA1-C3-A5 gene cluster among Asian Indians with premature CAD. However, our findings will benefit from the sequencing of this region in a small group of probands in order to understand the genetic variability of this highly heterogeneous chromosomal region in a population that does not have a representation, as yet, in the HAPMAP database. We plan to embark on this study in the near future. Additionally, we hope to confirm these preliminary, yet interesting observations, on a larger cohort of over 2000 CAD patients and age and gender- matched controls.

In conclusion, the Sac 1 and the -75G>A SNPs could serve as useful tools for the direct quantitative measure of variation in lipid and lipoprotein levels and an indirect assessment of CAD risk among the Asian Indian population.

## Abbreviations

ApoA: Apolipoprotein A; APOA1: Apolipoprotein A1 gene; APOA5: Apolipoprotein A5 gene; ApoB: Apolipoprotein B; APOC3: Apolipoprotein C3 gene; ASPs: Affected sibling pairs; BMI: Body mass index; CAD: Coronary Artery Disease; FCHL: Familial Combined Hyperlipidemia; HDL-c: High-Density Lipoprotein cholesterol; HTN: Hypertension; IBD: Identity By Descent; IBS: Identity By State. LDL-cholesterol: Low Density Lipoprotein cholesterol; LOD score: Logarithm of Odds score; MI: Myocardial Infarction; SAGE: Statistical Analysis for Genetic Epidemiology; SOLAR: Sequential Oligogenic Analysis Routine; SNPs: Single Nucleotide Polymorphism; TC: Total Cholesterol; TG: Triglyceride; Type 2 DM: Type 2 Diabetes Mellitus; UTR: Untranslated region; WHR: Waist Hip Ratio; QTL: Quantitative Trait Loci.

## Competing interests

The authors declare that they have no competing interests.

## Authors' contributions

JS was involved in the study design, molecular studies, data analysis, and drafting of the manuscript and takes full responsibility for the accuracy and integrity of the data. GP was responsible for carrying out the genotyping assays while NBK performed the lipid analysis. VSR supervised the data quality of the phenotypic assays and gave useful insights while preparing the manuscript. SJ carried out the statistical analysis while SH designed the association analysis and helped in data interpretation. MM conceptualized the study. VVK provided critical intellectual content while preparing the manuscript and gave the final approval for the publication of this article. All authors read and approved the final manuscript.
